# Regulatory Elements in Vectors for Efficient Generation of Cell Lines Producing Target Proteins

**Published:** 2015

**Authors:** O. Maksimenko, N. B. Gasanov, P. Georgiev

**Affiliations:** Institute of Gene Biology, Russian Academy of Sciences, Vavilova str. 34/5, 119334, Moscow, Russia

**Keywords:** insulators, recombinant proteins, protein production in mammalian cells, UCOE, S/MAR, STAR

## Abstract

To date, there has been an increasing number of drugs produced in mammalian cell cultures. In order to enhance the expression level and stability of target recombinant proteins in cell cultures, various regulatory elements with poorly studied mechanisms of action are used. In this review, we summarize and discuss the potential mechanisms of action of such regulatory elements.

## INTRODUCTION

Therapeutic proteins are major structural and regulatory molecules essential for a normal functioning of the human body. Part of recombinant proteins that are small in size and do not require additional modifications are produced in the most economical bioreactor –*E.coli* cells. However, the production of proteins in bacteria is associated with a number of limitations: disrupted folding of some proteins, absence of crucial modifications, and the inability to produce larger molecules [1, 2]. Some of these limitations are avoided when using yeast cells, which can produce high-quality recombinant proteins at sufficiently low cost. However, many recombinant human proteins require specific modifications that can only be obtained in higher eukaryotic cells [3]. Therefore, nowadays there is an increasing number of drugs obtained from mammalian cell cultures grown in bioreactors, mostly Chinese hamster ovary (CHO) cells [4, 5]. CHO cells were first isolated in 1957 [6]. It soon became evident that these cells are ideal for biomass scaling during the generation of recombinant proteins at the bioreactor level, since they are undemanding vis-a-vis growth conditions. Several lines were obtained from the initial clone of CHO cells, among which the CHO-K1 line became the most commonly used [7]. Optimal conditions for providing high growth density of CHO cells in bioreactors were selected using this cell line, enabling a significant increase in the product (target protein) yield alongside a reduced chance of human virus transmission [1, 4, 5]. Yet, the main problem in recombinant protein production in cultured cells is the extremely low product cost; therefore, there is a constant effort to reduce the expenses on obtaining high-producing cell cultures and elevate the yield of the protein product by enhancing the expression level of the target protein, cell culture density, and decreasing cell death. One of such approaches is vector improvement for obtaining transgenes, which can significantly reduce expenses in the generation of producing cell cultures. This paper presents an overview of the regulatory elements used in vector constructs for the generation of transgenic lines.

## VECTOR CONSTRUCTS FOR GENERATION OF TARGET PROTEIN-EXPRESSING CELL LINES

The most widely used method in industrial biotechnology is transfection with linearized plasmid DNA [3, 8], which allows one to obtain cell lines containing multiple copies of the expression vector that are usually integrated in one or, rarely, several genomic sites. The mechanism of vector construct integration into the genome has not yet been fully elucidated. Introduction of linear DNA into the nucleus results in the activation of reparation systems that provide cross-linking of linear DNA ends through two main mechanisms: homologous recombination and ligation of nonhomologous DNA ends [9-11]. As a result, long linear DNA molecules are formed bearing several copies of the vector construct capable of integrating into the genome with some probability. Linear DNA can integrate a genomic region that already contains other copies of the same DNA through the mechanism of homologous recombination, which leads to an increase in the copy number of the construct integrated in a specific genomic site [12]. Thus, one genomic site can bear up to several hundreds of integrated copies of a vector construct.

In order to enhance the producing capacity of clones, selective increase in the number of construct copies, which results in target protein expression, is used in industrial biotechnology [8]. This is achieved by decreasing the functional activity of the reporter gene encoding enzyme dihydrofolate reductase (DHFR), which catalyzes conversion of dihydrofolate to tetrahydrofolate and is essential for the synthesis of glycine, purines, and thymidine acid [13, 14]. DHF-auxotrophic cell lines can grow in media that necessarily contain glycine, as well as a source of purines (hypoxanthine) and thymidine. The derivative line CHO-DG44 with mutations of both alleles of the dihydrofolate reductase gene was obtained by random mutagenesis of the CHO-K1line quite a long time ago [15, 16]. It enabled to use dihydrofolate reductase as a selection gene for the generation of producing cell lines. Furthermore, in order to selectively increase the number of expression vector copies, which usually correlates with an increase in the target protein level, methotrexate (MTX) is used, which is able to selectively inhibit dihydrofolate to tetrahydrofolate conversion [2]. Treatment of transfected cell lines with MTX results in the survival only of cells with a significantly elevated level of dihydrofolate reductase. In most cases, this is caused by an increase in the copy number of the *dhfr* gene included in the construct and, as a result, of the gene encoding the target protein. Another frequently used selectable marker is the gene encoding glutamine synthetase (GS). The CHO-K1 cell line, which contains mutated allele of *gs*, is used when working with this marker. In this case, *L*-methionine sulphoximine (MSX) is used as a selective agent facilitating the selection of the most effective clones [2].

Clone selection can be conducted using other reporter genes, as well. The most promising options are the ones that do not require selection of a mutant cell line. Fluorescent protein technology, which allows one to select cells with maximum expression of a target gene based on emission at a particular wavelength, can be considered [17, 18]. Such an approach can be used for the generation of stable cell lines, which, in contrast to mutant derivatives deficient in *dhfr *and *gs*, exhibit a higher proliferative potential and viability. Among the disadvantages of using fluorescent protein genes as selectable markers is the inability to amplify the copy number, which in most cases would lead to increased producing capacity of a cell clone.

Recently, the application of special robots capable of selecting individual cell clones with the most efficient expression of the target protein, which is identified using antibodies, has become widespread [4]. Alongside with other advantages, this technology enables to avoid using markers the expression of which does not always correlate with the target protein level. ;

Vector constructs have also been developed based on viruses, mobile elements, bacterial anti-phage protection system, and recombination systems in phages and yeasts [3, 19]. Application of such vectors in several cases enables single insertions of a target gene into a specific genomic region, which is commonly used in gene therapy when generating transgenic cell lines and animals in order to obtain model systems for the study of gene expression regulation processes [20].

The main challenge in obtaining producing cell lines containing multiple expression vector copies is heterochromatin formation of repeated sequences of vector constructs, which usually enhances upon cell proliferation. The main role in heterochromatin formation of a repeated DNA sequence is played by RNA interference and noncoding RNAs that can stimulate repressive chromatin zone formation at promoters, as well as methylation of CpG sites in promoter regions, which decreases the efficacy of transcriptional factor binding in such regions [21-23]. This can result in a significant decrease in the target protein level after the obtainment of highly producing cell lines for some period. Moreover, heterochromatin formation at repeated copies of a vector construct can negatively affect the activity of adjacent cellular genes, which often leads to a decreased viability of producing cell lines. Repression of transcription from integrated repeats of a vector construct is caused by the cellular response to the introduction of foreign information, the expression of which should be suppressed, into the genome. Thus, application of regulatory elements capable of supporting efficient performance of the target protein gene alongside isolating regulatory elements of a vector construct from genomic regulatory elements seems to be extremely important.

## USE OF PROMOTERS AND ENHANCERS FOR GENERATION OF CELL LINES PRODUCING TARGET PROTEINS

Strong viral promoters, such as the cytomegalovirus (CMV) promoter and the early promoter of SV40, as well as strong cellular promoters of housekeeping genes, such as β-actin and factor EF1α genes, are usually used for transgene expression [24].

Strong viral or cellular promoters contain a minimal promoter of approximately 100 bp, which serves as the transcription start site (TSS), and a strong enhancer located in close vicinity to the promoter. For instance, the most widely used CMV promoter bears a core region located between positions –62 and –1 bp from TSS and the enhancer (–544 through –63 bp) [25]. There are several motifs in the region of the minimal promoter that determine the association with various components of the core transcription complex (TFIID): TATA [26], INR [27], DPE [28], BRE [29], DCE [30], and MTE [31]. However, such regions are not essential, since most strong promoters do not contain these elements or belong to the class of GC-rich promoters. One can assume the existence of a practically unexplored group of socalled architectural proteins determining the capacity of the core promoter to recruit the TFIID complex [32]. Unfortunately, the promoter architecture has not been studied deeply enough to determine, based on the sequence of a minimal promoter, its capacity to effectively bind the core transcription factors necessary for the performance of a strong promoter.

One of the approaches in applying effective promoters in biotechnology is identification of strong promoters directly in the cell cultures that are further to be utilized for the generation of target protein-producing lines. Thus, total genome screening of the strongest promoters in CHO cells that are most commonly used as the expression system in mammalian cells has been performed [33-35]. As expected, the most effective promoters appeared to be the promoters of housekeeping genes, including some ribosomal genes. However, the pitfall of this approach is a significantly high chance that the promoters identified by a genome-wide analysis may perform effectively only when located in a certain genomic region (the position effect) or when containing a complicated regulatory region, which significantly decreases the attractiveness of using such promoters in expression systems for obtaining target proteins from transgenic CHO cell lines.

One of the solutions to the position effect is using long regulatory sequences of actively transcribed housekeeping genes located on both sides of the coding region of the gene. Thus, a high expression level of target proteins (6–35 times the level of expression from a standard CMV promoter) has been obtained for vectors bearing a 12 kbp regulatory region or a 4 kbp 3’-region of the Chinese hamster *EF1*α gene [36]. One of the problems in utilizing long regulatory sequences for target protein expression is the instability of large vectors and decreased efficiency in generating multicopy lines that predominantly bear full constructs capable of expressing a target protein. The perspective model is the construct that includes long regulatory DNA regions from terminal repeats of the Epstein-Barr virus, which provides an order increase in efficiency in obtaining stably transfected cells [37].

Artificial modification of promoters is another promising approach to enhance their activity. For instance, a strong CMV promoter has been demonstrated to undergo negative regulation resulting in methylation of GC regions at transcription factor (TF) binding sites comprising promoters and, as a consequence, inhibition of TF recruitment to the promoter. As a result, the activity of the CMV promoter is greatly decreased. Such a negative affect can be avoided by integrating between the enhancer and core elements of the CMV promoter a regulatory sequence that binds transcriptional factors suppressing the DNA methylation process [38]. An effective promoter consisting of two divergent core elements with a single CMV enhancer integrated between them has been developed based on two CMV promoters [39]. This bidirectional promoter is able to express two divergent genes with approximately the same efficiency, which plays an important role in the production of proteins consisting of two different subunits (e.g., monoclonal antibodies).

A novel means to increasing a target protein expression is artificial recruitment of effective transcriptionassociated complexes to the promoter [40, 41]. For instance, histone acetyltransferase p300 binds active enhancers and promoters and also participates in the stimulation of transcription [42]. Recruitment of p300 to promoters significantly enhances efficiency in generating stable cell clones with a high level of a reporter gene expression [43]. It is worth mentioning that opposite results have been obtained in similar experiments with the Brahma remodeling complex, which provides increased mobility of nucleosomes and is capable of positively/negatively regulating transcription depending on a particular gene.

In order to enhance reporter gene transcription and reduce transgene expression dependence on the surrounding chromatin, strong cellular enhancers are used [44, 45]. One of the most frequently used enhancers is LCR (Locus Control Region), which controls the expression of human β-globin locus genes [46]. The main disadvantage of using enhancers for elevating transgene expression is due to their specificity, i.e. the ability to function only in certain cell lines, which imposes certain restrictions on their use as a general regulatory element. The search for the enhancers most efficient in the cell lines that are used for the generation of proteins at an industrial scale seems to be a promising trend in this direction [47].

## PROSPECTIVE USE OF INSULATORS FOR ENHANCING THE EFFICIENCY OF TARGET GENE EXPRESSION IN PRODUCING CELL LINES

In order to increase the efficiency and stability of target protein expression, known insulators are used [41, 48-51]. Insulators are regulatory elements that block interaction between the enhancer and promoter if interposed between them [52, 53]. In addition, insulators do not directly affect an enhancer’s and promoter’s activity, which means that the promoter can be activated by any other enhancer, and the enhancer, in its turn, is capable of activating any other promoter. In addition, some insulators can serve as boundary elements between transcriptionally active chromatin and heterochromatin. The best-studied examples are insulators of fruit fly Drosophila and vertebrates. Initially, it was assumed that insulators determine the borders of transcriptional domains within which gene expression does not depend on the negative effects of the surrounding genome [54, 55]. However, it has been later demonstrated that insulator proteins are considerably more flexibly integrated into the gene regulatory system [52, 53].

Recently, certain insulator proteins have been shown to participate in the organization of specific, long-range interactions between distal regions of chromatin [56-59]. Insulator proteins can support interactions between enhancers and promoters, boundaries of transcriptional domains which are usually located up to several hundreds of kbp away [34, 60-62]. The obtained results allowed one to refer the class of insulator proteins to chromatin architectural proteins [32, 53].

**Fig. 1:**
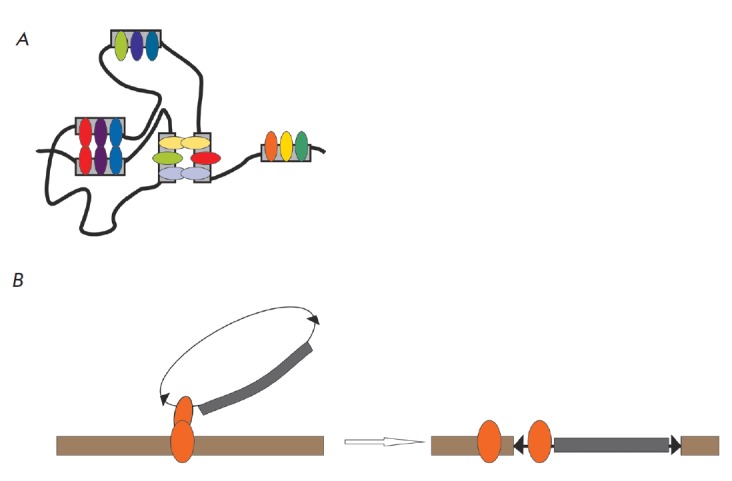
*A *– Model of establishing specific long-range interactions between regulatory elements. Several IAP (insulator architectural proteins) bind with each element. As a result, two identical elements are capable of providing highly specific and efficient ultra long-range interactions between regulatory elements, but only weak contacts are formed upon partial overlap of IAP-binding sites in regulatory elements. Grey rectangles represent regulatory elements binding with IAP; colored ellipses depict combinations of IAP that specifically bind their own sites and interact with each other. *B *– Specific integration of a transgenic construct into a particular genomic region. The transgenic construct is presented as a circle. Black triangles correspond to P element end repeats; grey rectangle, –elements comprising a transgenic construct. Orange ellipses –regulatory elements that bind with IAP. Brown rectangle – a genome region

To date, insulator architectural proteins (IAP) of Drosophila remain poorly described, which is largely due to the ease of generating transgenic lines of flies. The study of insulator properties in Drosophila transgenic lines showed that each insulator binds several IAP, which in turn determine the specificity of longrange interactions [32, 53]. As a result, two identical insulators can provide sufficient specific ultra longdistance interactions between regulatory elements in Drosophila transgenic lines [63, 64], which allowed researcher to propose a model in which IAP associated with specific binding sites, comprising regulatory elements, create a code, which in turn determines how effective a long-range interaction established between these regulatory elements will be (*Fig. 1A*) [32].

Usually, transgenic Drosophila lines are obtained by injecting a vector that contains P element ends flanking the transgenic construct alongside the gene encoding transposase essential for construct integration into the genome [65]. Cases have been described of highly specific integration of the P element that includes an insulator or a strong promoter into a certain genomic region containing the cognate endogenous regulatory element [66, 67]. Such highly specific integration of the P element called *homing *can be explained by the recruitment of IAP to the regulatory element that comprises the P element upon introduction of the construct into an embryo. This results in specific interaction between the P element and the cognate endogenous regulatory element, with further integration of transposon into a certain region at the chromosome (*Fig. 1B*).

Genome-wide studies of IAP-binding site distribution unambiguously demonstrated that insulators are not fixed boundaries between transcriptional domains [62, 68]. Two mechanisms of enhancer activity suppression by insulators have been described in Drosophila transgenic lines, cell cultures, and *in vitro *[53]. The first mechanism is based on the appearance of topological obstacles that block interactions between the enhancer and promoter. As a result of the formation of stable contacts between insulators, one of the interacting regulatory elements appears to be isolated in the independent chromatin loop. Such a mechanism of insulator action was found using artificial insulators that contained binding sites for proteins capable of effective interaction with each other and formation of stable chromatin loops [69, 70], and using transgenic lines of Drosophila [71]. This mechanism manifests itself effectively only in the case when insulators are immediately adjacent to the suppressed elements (enhancers and promoters). When the size of the chromatin loop formed by insulators is larger, such an enhancer blockage pathway is not utilized [72].The second, more common, mechanism is based on the establishment of direct contacts between the proteins associated with the insulator and enhancer-promoter complex. For example, it has been demonstrated in transgenic lines of Drosophila that insulators may directly interact with promoters and enhancers [71, 73]. In such a case, when the insulator is located between the enhancer and promoter, insulator proteins interfere with the establishment of proper contacts between the transcriptional complexes assembled at the enhancer and promoter, which leads to partial or complete inability of the enhancer to stimulate transcription from the promoter.

The mechanisms that determine the barrier function of insulators have been described in detail in Drosophila and mammals. In particular, it was found that IAP help to recruit the protein complexes responsible for nucleosome remodeling and modification to insulators [74-78], resulting in the formation of open chromatin zones. At the same time, some insulators can recruit the protein complexes directly involved in transcription stimulation [75]. Due to the formation of nucleosomefree DNA regions and recruitment of transcriptional complexes, insulators suppress the spread of repressive chromatin, which, nevertheless, does not exclude the possibility of direct interaction between insulators and silencers initiating heterochromatization.

To date, only a single DNA-binding insulator protein, CTCF, has been described in vertebrates [79, 80], which is probably due to the absence of convenient model systems for the study of insulators. CTCF is capable of supporting long-range interactions between distal areas of chromatin [60, 79-81]. Thus, CTCF is the first architectural protein characterized in a mammalian genome [32, 80].

Except for their key role in the formation of chromatin architecture, CTCF protein domains remain poorly studied and the mechanism of CTCF performance in maintaining long-range interactions has not been characterized yet. The main part of the protein consists of 11 C2H2-type zinc fingers (ZF), with only four of them (4th to 7th) being essential for the recognition of the core DNA motif [82]. The remaining zinc fingers seem to recognize the specific nucleotide sequences stabilizing CTCF association with DNA. The most logical suggestion is that a protein supporting long-range interactions is capable of effective di- and multimerization. Indeed, CTCF has been shown to be able to homodimerize; however, the domain responsible for this activity has not been identified yet [83]. Evidence has been obtained that the C-terminal domain of CTCF also interacts directly with its own zinc fingers [84]. However, such interaction cannot be highly specific, because CTCF zinc fingers bind many other transcription factors, as well: CHD8, Sin3A, and YB-1 [85-87].

The cohesin complex, which associates directly with CTCF [90], is suggested to play a significant role in the organization of long-range interactions [60, 80, 88, 89]. The cohesin complex is recruited to chromatin by CTCF and facilitates the formation of long-range interactions between CTCF genomic sites. This model is consistent with genome-wide studies demonstrating a high degree of colocalization of CTCF and cohesin subunits [91, 92]. However, a very slight decrease in binding of cohesins to chromatin has been shown in experiments on CTCF inactivation, suggesting the implication of other transcription factors in the recruitment of the cohesin complex at chromatin [92-94]. Moreover, inactivation of CTCF and cohesins leads to various disruptions of the chromatin architecture [95, 96], which can be attributed to independent functioning of these proteins.

**Fig. 2:**
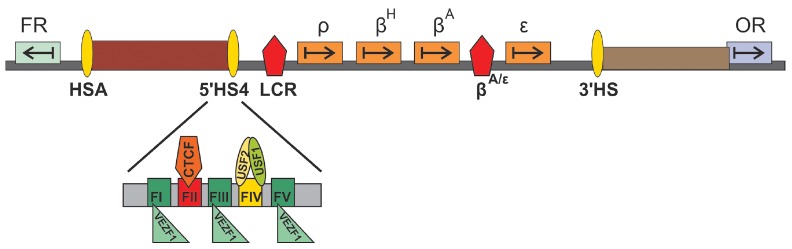
Schematic representation of β-globin locus and adjacent sequences**. **Designations ρ, β^H^, β^A^, ε correspond to the genes β-globin locus; FR – folate receptor gene; OR – olfactory receptor gene. Arrows indicate the direction of gene transcription. HSA, 5’HS4, 3’HS – insulators; LCR, βA/ε – enhancers of β-globin locus. Insulator HS4 is represented schematically in detail. FI, FIII, FV –binding sites for protein Vezf1/BGP1. FII and FIV – protein CTCF- and heterodimer USF1/2-binding sites, respectively

The most well-studied vertebrate insulator, HS4, consisting of 1,200 bp and located at the 5’-end of the chick β-globin locus, is used in biotechnology (*Fig. 2*) [97, 98]. A core region of 250 bp has been found in this insulator, which exhibits the activity of the complete insulator and contains five fragments (FI, FII, FIII, FIV, FV), each of which has its own functional value. A site that binds CTCF, which is necessary and sufficient for the manifestation of the enhancer-blocking activity of HS4, has been identified in the FII region of the insulator [99]. Proteins USF1 and USF2, which bind as heterodimers to the FIV region, are responsible for boundary formation between active chromatin and heterochromatin [100]. USF has been shown to recruit the protein complexes responsible for modification of the histones associated with transcription stimulation [100, 101]. The protein BGP1/Vezf1, which possesses a DNA-binding domain consisting of zinc fingers, associates with other regions of the HS4 insulator (FI, FIII, FV) [102]. The protein BGP1/Vezf1 protects GC-rich regions of the insulator from methylation, which affects the recruitment of insulator proteins to DNA and, therefore, results in insulator inactivation. According to the existing model, BGP1/Vezf1 terminates weak transcription from the region of heterochromatin, which may play an important role in protection of the β-globin locus from the spread of inactive chromatin [103].

Since its discovery, insulator HS4 has been actively utilized for transgene expression in mammalian cell cultures [98]. Two complete copies of HS4 have been integrated into a vector for producing transgenic animals expressing a target protein in milk [104]. It was demonstrated that the insulator substantially enhances the expression of target proteins, but it has no significant affect on the specificity of transgene expression only in the mammary gland, and does not provide a direct correlation between the copy number of the construct and the level of target protein production [105].

Most effectively, HS4 insulator can be applied in vectors that for some reason have a limited size. Thus, a full-size 1.2 kbp insulator significantly reduces the efficiency of cellular transformation with lentiviral vectors (probably due to the limitations imposed on the size of the viral particle). Therefore, HS4-duplicated core element of 250 bp, which contains the binding sites of all the identified transcription factors required for the manifestation of insulator activity, is used in vectors of this class [98, 106]. Insulators are also successfully used for protecting reporter gene expression in vectors designed based on mobile elements [51] and retroviruses [107].

Despite the examples of successful use of a 1.2-kbp HS4 insulator or its core region [98, 108], abundant data have been obtained showing that HS4 does not have a positive effect on target gene expression. This can be explained by the fact that the cell cultures that were used in the experiments differed significantly in the set of transcription factors that bind with the HS4 insulator.

In conclusion, the following basic mechanisms for the protection of transgene expression using the HS4 insulator can be put forth: 1) formation of a nucleosome- free DNA region that can disrupt the linear spread of heterochromatin; and 2) recruitment of protein complexes that enhance nucleosome mobility, modify histones, stimulate transcription, protect CpGsites from methylation, and terminate weak transcription. It has not been determined yet whether the HS4 insulator is capable of guiding construct integration into transcriptionally active chromatin zones and directly interacting with the target gene promoter for further transcription stimulation. Apparently, the main disadvantage of HS4 and other insulators is the dependence of their activity on the set of particular transcription factors expressed in the cell line.

## ENHANCING TRANSGENE EXPRESSION USING A/T-RICH SEQUENCES ASSOCIATED WITH NUCLEAR MATRIX PROTEINS (S/MAR)

In order to enhance transformation efficiency and improve the stability of transgene expression, sequences of 300-5,000 bp, usually A/T-rich, which interact with a fraction of the nuclear matrix (S/MAR, scaffold matrix attachment region), as shown in experiments* in vitro*, have been widely used from the beginning of the 90s [108-110]. S/MAR regions possess a number of distinctive properties: they are A/T-rich regions, sensitive to DNase I, and potentially tend to form left-handed helix and triplex structures [111, 112]. It is assumed that it is the A/T-rich composition of these elements which leads to the destabilization of the double helix and ability of MAR to generate areas rich in various secondary structures [113, 114].

Based on the characteristics of the secondary structure, there is about an order of 50,000 elements predicted for the human genome that supposedly share the properties of S/MAR [115, 116]. A total of 1,500 regions that have the most relevant characteristics of S/ MAR have been selected from this pool. Only several of them turned out to share a high level of homology with mouse orthologs, implying that the nucleotide sequence of S/MAR regions lacks distinctive, conserved elements.

The structure of S/MAR elements indicates that they might serve as recombination hotspots. Indeed, it was shown that disruption sites that occur due to inversions associated with human diseases are often localized in S/MAR-elements [117, 118], and integration of retroviruses into the genome occurs in close vicinity to S/MAR at a high frequency [119, 120]. According to some reports, S/MARs participate in the regulation of DNA replication [121-123]. It was found that S/MAR elements enhance transgene expression and reduce expression variability during the generation of stable cell lines [41, 124]. Expression of a gene surrounded by S/MAR elements has been experimentally established to be proportional to the gene copy number [125]. It is assumed that S/MAR elements can be functionally regarded as insulators that protect transgene expression from the positive/negative effects of the surrounding chromatin.

Initially, it was thought that lamina are a major component of nuclear matrix proteins [126]. Later, many other additional proteins, including transcription factors, were found in the nuclear matrix [127]. S/MAR often contain the binding sites of such transcription factors as SATB1, Fast1, CEBP, SAF-A, and SAF-B (proteins that preferentially bind to A/T-rich regions), NMP4 (matrix protein), CTCF and Hox family proteins [83, 116, 128-131]. Topoisomerase II also predominantly associates with A/T-rich regions within S/MAR [132-134]. Reduced density of nucleosome distribution in S/MAR elements and increased concentration of histone acetylation complexes is explained by the association of numerous transcription factors and ability of these elements to form secondary structures.

SATB1 is the most well-studied matrix protein involved in many biological processes, such as differentiation of T cells and epidermis [135-137]. SATB1 can be included in the class of architectural proteins capable of maintaining specific long-range interactions [135]. SATB1 forms homodimers and binds to A/T-rich sequences with two CUT domains and one C-terminal homeobox. Apart from participation in chromatin domain formation, SATB1 recruits ASF1 (ATP-dependent factor involved in chromatin organization) and the ISWI complex (enhances nucleosome mobility) [128, 138].

SAF-A, another matrix protein, includes a DNAbinding (SAF) and an RNA-binding (RGG) domain. It is interesting that Xist RNA, which regulates dosage compensation in mammals, also associates with the RGG domain. This interaction determines the localization of Xist RNA on the X chromosome [139]. According to the existing model [140], SAF-A recruits Xist RNA to a S/MAR element located in the region of initiation of heterochromatin formation on the X chromosome. Interaction between the proteins SAF-A and SATB1 further results in the formation of a loop between neighboring S/MAR complexes, which ultimately leads to the spread of Xist RNA on chromosome X and its subsequent inactivation.

According to the most commonly used model, S/MAR elements interact with the proteins of the nuclear skeleton (matrix proteins), resulting in the formation of chromatin loops where S/MAR serves as a core element [141]. Genes located within a chromatin loop formed by S/MAR are assumed to be protected from the negative influence of the surrounding chromatin [109]. Nevertheless, the structure of the nuclear matrix and role of S/MAR in the organization of the chromosome architecture still remain elusive. According to recent concepts, the matrix presents labile conglomerates of proteins, which transiently interact with S/MAR protein complexes comprising the chromosomes [141].

Despite the lack of understanding of the mechanisms underlying S/MAR action, abundant experimental data has been obtained demonstrating the effectiveness of using these elements for enhancing the expression of target proteins in mammalian cell cultures [142]. For example, S/MAR of the lysozyme gene from the chicken egg causes a 5- to 10-fold increase in the level of monoclonal antibody expression in CHO cells [109, 143]. Later, other, more effective mammalian S/MARs were characterized [144]. S/MARs have been successfully used to increase the expression level of erythropoietin, as well as human growth factor TGF-β receptor type II [108]. Furthermore, S/MAR appears to function both within viral vectors [145] and vectors designed based on transposable elements [146]. S/MAR effectively protects transgene expression from repression (barrier activity) and also supports a higher level of transcription from promoters within expression vectors (stimulatory activity) [144]. Several S/MARs are capable of increasing the efficacy of vector construct integration into chromosomes [147, 148]. Moreover, S/MAR proteins are able to provide integration of new additional copies of the vector construct in a region already containing integrated construct copies. The best possible explanation for such a characteristic of S/MAR is that the interaction between architectural proteins (such as SAF-A and SATB1) provides a contact between S/MAR copies located in the genome and plasmid (analogue of the homing phenomenon in Drosophila mentioned above). Apparently, the increased recombination activity provided by S/MAR elements enhances the efficiency of transgene integration into specific regions of the genome. Thus, the positive impact of S/MAR on transgene expression might be largely determined by directed integration of a vector construct into normally transcriptionally active S/MAR-containing areas. This implication is consistent with the finding that many of the studied S/MARs have a positive influence on gene expression only when integrated into the genome [125, 144]. In order to increase the amplification rate of a construct in cell clones, S/MAR was combined with the mammalian replication initiation region [149, 150]. Treatment of primary transfectants with MTX enabled to achieve large-scale amplification of a construct in cell clones, which led to a stable multifold increase in the production of the target protein [150].

In conclusion, it can be stated that S/MAR present regulatory elements that are less studied than insulators. The most probable mechanisms of S/MAR action in enhancing transgene expression are 1) site-specific integration of S/MAR-bearing constructs into the regions of transcriptionally active chromatin and amplification of the copy number of the integrated construct, 2) association with S/MAR complex elements that guide transcriptionally active chromatin zones and thus suppress the spread of heterochromatin, and 3) immediate promoter activation by transcription factors that directly bind to S/MAR.

## ENHANCING TRANSGENE EXPRESSION IN CELL CULTURES BY REGULATORY ELEMENTS BEARING A STRONG PROMOTER OF HOUSEKEEPING GENES

Between 2000 and 2002, a small company named CobraTherapeutics developed a technological platform based on regulatory elements isolated from housekeeping genes, which are actively transcribed at all stages of development and in all cells of an organism, for obtaining efficient cell lines producing recombinant proteins [45]. These regulatory elements were named ubiquitous chromatin opening elements (UCOE) since the promoter regions of the actively transcribed genes are characterized by a low density of nucleosomes, which is due to the presence of DNA-binding TF stimulating transcription. The best characterized UCOE are DNA regions that contain a pair of divergent gene promoters, *HNRPA2B1* and *CBX3 *or *TBP *and *PSNB1*, which are actively transcribed in all cells of an organism [151]. The first experiments used large regulatory elements of 12-16 kbp, which significantly increased the percentage of transfected cells and provided high-level and stable transgene expression for a long cultivation period [151, 152]. Thus, UCOE causes a 16-fold increase in the efficiency of the CMV promoter, which is highly susceptible to inhibition by RNA interference and methylation of CpG regions [152, 153]. It was shown that UCOE can maintain a high expression level of a transgene integrated into pericentromeric heterochromatin. UCOE are also effective as part of lentiviral vectors [154-159]. It can be assumed that UCOE are bound by transcription factors that recruit the complexes preventing methylation of CpG repeats and forming chromatin areas with reduced nucleosome density in the promoter regions comprising lentiviral vectors [155, 160].

It should be noted that, unlike other regulatory elements such as LCR and enhancers, which exhibit pronounced cell specificity, promoters of housekeeping genes can function effectively in various cell lines. In experiments with various UCOE that were reduced in size in order to assess the possibility of using UCOE in expression vectors, more compact-size variants of UCOE (1.5 to 3 kbp) have been obtained. Such truncated elements completely retain their activity during the generation of high-producing cell lines [152].

UCOE actively participate in the process of transcriptional regulation, which implies the existence of direct interactions between the promoter regulatory elements responsible for the expression of the reporter gene located in the vector and the transcription factors associated with UCOE. Therefore, UCOE can effectively act only on certain promoters and the functional activity of these promoters does not manifest itself in cell lines [161]. Some studies have demonstrated that UCOE themselves can be used as promoters for providing stable expression of a reporter gene [162]. However, contribution in transgene expression of the transcription initiated from promoters comprising UCOE is ambiguous, and its role remains elusive. In particular, there are experimental data showing that UCOE do not always effectively enhance the expression of a target protein in CHO cells [162, 163]. Negative results obtained using UCOE can be explained by the fact that strong promoters comprising UCOE induce transcription that in some cases is capable of triggering RNA interference and/or recruiting repressive complexes to the promoter that transcribes the reporter gene.

It was also demonstrated that a combination of two strong promoters may in some cases facilitate the generation of stable cell lines and enhance transgene expression [164]. Analysis of various combinations of two of the promoters CMV, SV40, RPL32, EF1-α and β-actin showed that only the *RPL32 *promoter, integrated before any other of the studied promoters, can significantly increase the efficiency of stable cell clone selection. It is worth mentioning that the direction of the *RPL32 *promoter should coincide with the direction of the promoter responsible for reporter gene expression, and core elements of the *RPL32 *promoter in this case are essential components of the system for providing a stimulating effect.

In general, combining strong promoters is a promising way to improve the efficiency of generating cell clones producing a target protein. Strong promoters bearing a combination of enhancer and core promoter recruit protein complexes, which in turn support a transcriptionally active state of chromatin. According to the data of genome-wide studies, promoters act as effective boundaries that are able to protect genome areas against the spread of repressive chromatin regions [165, 166].Transcription factors that bind to properly matched promoter pairs can mutually reinforce each other’s activities. Apparently, the use of some promoters can provide advantageous integration of a construct into certain chromosome areas with the highest levels of transcription. A more complete understanding of the mechanisms of transcription activation will further allow researchers to modify promoters in order to improve their performance when using them in expression systems.

## ENHANCING THE TRANSGENE EXPRESSION LEVEL IN CELL CULTURES BY REGULATORY ELEMENTS PROTECTING FROM HP1-DEPENDENT REPRESSION

The Chromagenic company has developed a technological platform based on a test-system which effectively allows the identification of regulatory elements capable of suppressing the spread of heterochromatin areas [45]. The test-system is based on the recruitment of the HP1 protein, which is responsible for heterochromatin formation, to a plasmid using the DNA-binding domain of the Lex protein [167]. The chimeric protein HP1-Lex binds to Lex-specific sites on the plasmid and recruits other components of the heterochromatin complex, which launches the inactivation of the adjacent promoter. This results in repression of *zeoR*, which is responsible for resistance to the antibiotic Zeocin, and the death of transfected cells when cultured in a selective medium with the addition of Zeocin. The screening aimed at detecting DNA fragments, integration of which between Lex binding sites and the *zeoR* promoter protects the promoter from HP1-dependent repression, enabled to find a series of regulatory elements 500 to 2,000 bp in length called antirepressors (STAR). Other known regulatory elements such as the insulator HS4, MAR, and UCOE lack that ability. A comparative analysis of various regulatory elements [165] has shown that STAR elements are most effective when using them for generating high-producing CHO cell lines. However, the mechanism of STAR element action remains unexplored. There is still no evidence on what transcription factors bind with elements of this class and provide them with functional activity.

## CONCLUSIONS

To date, no universal regulatory element with a clear mechanism of action has been found that can be effectively used in all types of vector constructs designed to generate cell lines producing various proteins at a high level. This is largely due to the complexity of the mechanisms that regulate promoter activity and also the absence of actual evidence on the original concepts of strict organization of genes with the same expression profile into transcriptional domains surrounded by special regulatory elements from a class of insulators or S/MAR. Clearly, some mechanisms must exist that suppress excessive transcription even for the strongest promoters. RNA interference is one of such mechanisms. It is possible that a detailed understanding of the mechanisms of transcription activation and suppression will lead to the development of artificial promoters that allow researchers to obtain stable high levels of target gene expression in transgenic systems.

## Abbreviations

RP: recombinant protein
CHO: Chinese hamster ovary cells
UTR: untranslated gene region
kbp: a kilobase pair, a unit of length of nucleic acids equal to 1,000 base pairs
S/MAR: DNA sequences corresponding to nuclear matrix-attachment regions; insulators – regulatory elements that block interaction between enhancer and promoter
UCOE: regulatory elements containing strong promoters of housekeeping genes
STAR: regulatory elements protecting from HP1-dependent repression
MSX: L-methionine sulphoximine
MTX: methotrexate
